# Using Hierarchically Structured, Nanoporous Particles as Building Blocks for NCM111 Cathodes

**DOI:** 10.3390/nano14020134

**Published:** 2024-01-06

**Authors:** Werner Bauer, Marcus Müller, Luca Schneider, Marcel Häringer, Nicole Bohn, Joachim R. Binder, Julian Klemens, Philip Scharfer, Wilhelm Schabel, Helmut Ehrenberg

**Affiliations:** 1 Institute for Applied Materials (IAM), Karlsruhe Institute of Technology (KIT), Hermann-von-Helmholtz-Platz 1, 76344 Eggenstein-Leopoldshafen, Germanyhelmut.ehrenberg@kit.edu (H.E.); 2 Thin Film Technology (TFT), Karlsruhe Institute of Technology (KIT), Straße am Forum 7, 76131 Karlsruhe, Germanywilhelm.schabel@kit.edu (W.S.)

**Keywords:** battery, lithium-ion battery, active material, cathode, porosity, nanomaterial

## Abstract

Nanoparticles have many advantages as active materials, such as a short diffusion length, low charge transfer resistance, or a reduced probability of cracking. However, their low packing density makes them unsuitable for commercial battery applications. Hierarchically structured microparticles are synthesized from nanoscale primary particles by targeted aggregation. Due to their open accessible porosity, they retain the advantages of nanomaterials but can be packed much more densely. However, the intrinsic porosity of the secondary particles leads to limitations in processing properties and increases the overall porosity of the electrode, which must be balanced against the improved rate stability and increased lifetime. This is demonstrated for an established cathode material for lithium-ion batteries (LiNi_0.33_Co_0.33_Mn_0.33_O_2_, NCM111). For active materials with low electrical or ionic conductivity, especially post-lithium systems, hierarchically structured particles are often the only way to produce competitive electrodes.

## 1. Introduction

A standard lithium-ion battery electrode consists of a particulate active material, conductivity enhancing components such as carbon black or graphite, and a polymer binder that holds all the particles together and attaches them to the metal current collector. The active materials play a central role in this concept, acting as a source and sink for the lithium ions to provide the desired capacity for energy storage. The theoretical capacity of the battery is determined by the chemical composition and the crystalline phase of the active material. However, the size, crystalline structure, and morphology of the particles also play important roles in the practical capacity. This is mainly because the diffusion of the ions in the active materials in the solid state is much slower than the transport rates in a liquid electrolyte. To improve the performance of the cells, various tailor-made structures have been developed [1], such as preferentially oriented crystals within the particles [2], nanobrick morphology [3], one-dimensional hierarchical microrods [4], and hierarchically structured particles [5,6,7,8].

Especially for high power densities, it is advantageous to use small particles that keep the diffusion distance in the solid state short and allow the lithium ions to enter the electrolyte as quickly as possible. Rapid ion transport reduces the occurrence of overvoltage at higher current densities. As a result, the cutoff voltage is reached later, resulting in a higher charge or discharge capacity compared to large active material particles. Although the larger specific surface area of smaller particles must not improve the rate performance by increasing the contact area between the electrolyte and the active material [9], it can multiply the contact points to the conductive carbon network. This improves the electrical connection, especially for active materials with low electronic conductivity. In addition, the mechanical integrity of active materials is known to improve with decreasing primary particle size. The stresses that occur during cycling accumulate in larger particles, making nano-sized active materials less susceptible to cracking than their macro-sized polycrystalline counterparts [10]. All these arguments support the use of nanoscale active electrode materials. In contrast, most active materials are commercially offered as dense, compact, and equiaxed particles in the 5–10 µm size range, ignoring the advantages of nanoparticles.

The main advantage of micron-sized active materials is the improved electrode packing, which enables high energy density at the cell level, while nanomaterials have a significantly lower loading. This is due to the need to add conductive additives and binders, otherwise the electrical conductivity or mechanical stability of the electrode is not suitable for battery applications. Carbon black, as the main conductive additive, typically has an average particle size in the nanoscale. In TEM studies, primary particle diameters are found to be less than 50 nm [11]. However, the primary particles form highly sintered aggregates with average sizes of 100–300 nm, which cannot be further disaggregated even by intensive mixing [12]. If the size of the active materials is of the same order of magnitude, the formation of a conductive microstructure is significantly hindered. Percolation theory can be used to describe this phenomenon [13]. It explains why the carbon black content must be 16 vol% or more for similarly sized particles, because only then will the statistically distributed carbon black particles form a percolation structure that ensures sufficient electrical connection of the active material particles to the current collector. On the other hand, if the active materials have a significantly larger particle size than the carbon black, the volume already occupied by the active material leads to directed percolation due to the localization and denser packing of the carbon black particles in the pores. As a result, the percolation threshold is lowered, and high electrode conductivity is achieved at lower carbon black levels. A particularly low percolation threshold is possible when there is a two-dimensional distribution in which the carbon black particles occupy only the surface of the active materials. In addition, if the carbon black and active material particles are the same size in the nanoscale, the number of particles increases massively. This also increases the amount of binder required to crosslink the particles. While nanoscale active materials require the addition of more than 20% *w*/*w* binder and conductive carbon black, the quantity of inactive components can be reduced to less than 5% *w*/*w* for common particle sizes of about 10 µm. The significantly higher active material content results in a higher energy density and explains why nanomaterials have not yet been widely used on an industrial scale.

## 2. Hierarchically Structured Electrode Materials

**Figure 1:**
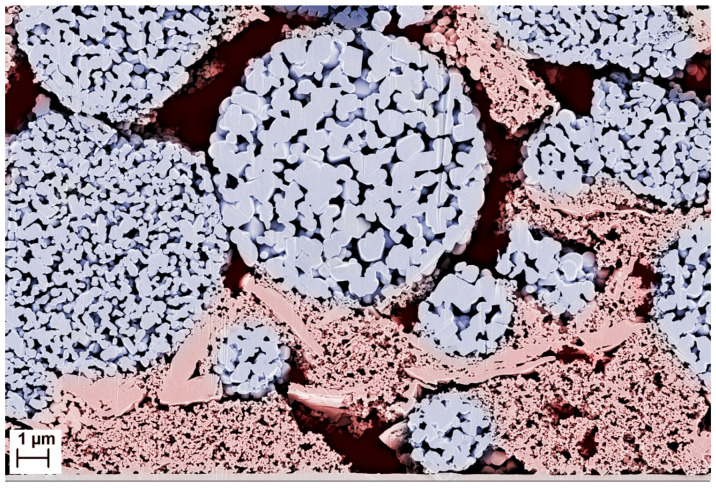
SEM cross-section through a hierarchically structured NCM111 cathode with regions of interparticular (red) and intragranular (blue) porosity. Interparticular pores are partially filled by binder-stabilized domains of the conductive additives carbon black and graphite.

Active materials with an easily accessible, nanoscale pore structure are an attractive approach to make the positive properties of nanomaterials available with microscopic particles or to achieve higher packing densities even when using nanomaterials [14]. For this purpose, the nanoparticles are agglomerated into microscopic granules. They are then thermally solidified by forming sinter bridges to produce particles that are stable enough to be processed into electrodes and cells analogous to conventional active materials. However, the sintering process must be limited to preserve the open pore structure of the agglomerates and nanoscale dimensions of the primary particles. This allows the electrolyte to fill the open pores and accelerate ion transport through the pore channels. In this state, the nanoporous particles form a hierarchical structure of interparticular and intragranular pores in the electrode (Figure 1), giving the materials their name.

The granulation step can produce both single-phase materials and composite materials. These composites can include an electrically conductive phase, allowing for the creation of active material powders with low electrical conductivity at high packing densities, which would otherwise only work at the nanoscale. This makes nanocomposites a prerequisite for certain materials to have commercial potential in batteries (also see chapter 7).

**Figure 2:**
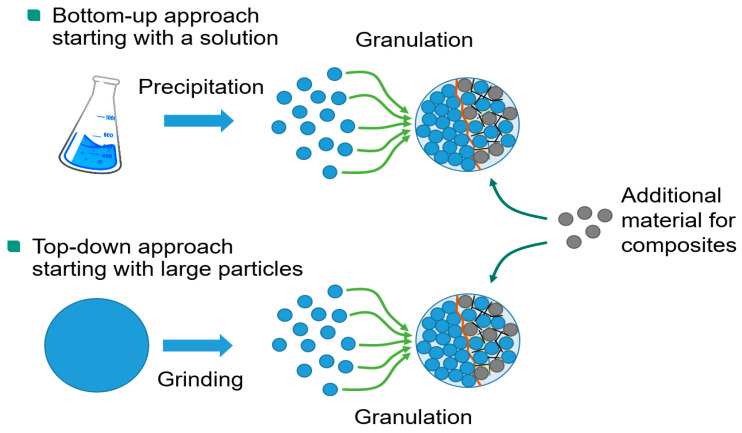
Schematic preparation of hierarchically structured cathode materials by a bottom-up versus a top-down approach.

Hierarchically structured materials can be prepared in two ways (Figure 2). In the top-down approach, a commercial electrode material with a micron particle size is wet-milled down to the nanoscale. Agitator bead mills have proven to be very efficient in achieving high particle fineness even at high throughputs. The addition of a dispersing agent helps to stabilize the resulting fines against re-agglomeration. The grinding suspension can be used directly in a spray dryer for the granulation step without intermittent drying process [6]. The alternative bottom-up approach starts with a precursor solution of acetates, carbonates, gluconates, or other salts of the desired cations [12]. The use of water as a solvent allows for a cheaper and more environmentally friendly synthesis. The nanoparticles are also precipitated from this solution by spray drying. The most elegant way is to combine the precipitation step with the subsequent granulation step and perform both processes in a single pass in the spray dryer [13]. However, this is only feasible for single-phase materials, while for composites it is necessary to separate the precipitation and granulation steps. The size of the granules can be adjusted from a few microns to over 30 µm by changing the speed of the impeller. This makes it possible to cover the entire range in which active materials are commonly used. Granulation is followed by a calcination step in which the precipitated granules are thermally treated in a furnace. This process removes unwanted phases and precursor residues, and heals mechanical damage caused by milling. The thermal treatment also induces the formation of the sinter necks between the primary particles, which increase the mechanical stability and electronic conductivity of the granules.

The bottom-up approach offers greater freedom in composition and morphological design, as a variety of precipitation reactions can be used. It also allows the synthesis of active materials that are not commercially available. It is the more economical approach because it does not require the costly milling step. The top-down approach is particularly interesting from a scientific point of view, as it allows a direct comparison of the properties of the dense starting material and the porous granules. In this paper, the processing behavior and electrode properties of dense and porous particles with similar sizes and chemical compositions are investigated for LiNi_0.33_Co_0.33_Mn_0.33_O_2_ (NCM111) using a top-down approach. NCM111 is less sensitive to moisture than NCM materials with higher nickel contents. This reduces the likelihood that the particle properties will be differently affected by reactions with the environment.

## 3. Experimental

### 3.1. Material Synthesis

For the top-down approach, LiNi_0.33_Co_0.33_Mn_0.33_O_2_ (NCM111) powder (NM-3100, Toda Kogyo Corp., Tokio, Japan) was suspended in deionized water with a dispersant (Darvan 821A, R.T. Vanderbilt, Norwalk, CT, USA) and milled in an agitator bead mill (LabStar LS1, Netzsch GmbH, Selb, Germany) with ZrO_2_ beads (0.1 mm or 0.2 mm diameter) at 3000 rpm for 1 h. The suspension was then spray-dried (MobileMinor spray dryer, GEA AG, Düsseldorf, Germany) with adjusted impeller speed to receive granules with nearly the same diameter as the original material. The spray-dried NCM111 granules were calcined at temperatures between 700 and 1000 °C for 5 h under air to yield a hierarchically structured cathode material.

The size of the secondary particles was determined via laser diffraction (Horiba LA950, Retsch Gmbh, Haan, Germany), surface area through N_2_ adsorption with evaluation by Brunauer-Emmett-Teller (BET) theory (Gemini VII 2390, Micromeritics Instruments Corporation, Norcross, GA, USA), and granule porosity and pore size distribution were measured by mercury intrusion porosimetry (CEI Pascal 1.05, ThermoElectron S.p.A., Milan, Italy).

### 3.2. Electrode Manufacturing and Testing

Cathodes for coin cells were prepared by mixing the hierarchically structured NCM111, a PVDF binder (KYNAR Powerflex LBG-1, Arkema, Lyon, France), and carbon black (C-NERGY Super C65, Imerys Graphite & Carbon, Bironico, Switzerland) with a magnetic stirrer in an 8:1:1 ratio in NMP and manually doctor-blading the slurry at a 200 µm gap height on an aluminum current collector. The foils were dried overnight at 80 °C and processed to half cells without calendering.

For pouch cells, NCM111 (as received or processed in the top-down approach) was mixed with C65 carbon black, graphite (C-NERGY KS6L, Imerys Graphite & Carbon, Bironico, Switzerland), and PVDF (Solef 5130, Solvay S.A., Bollate, Italy) in N-methyl-2-pyrrolidone (NMP, Carl Roth Gmbh, Karlsruhe, Germany) using a dissolver mixer (Dispermat SN-10, VMA Getzmann GmbH, Reichshof, Germany) for 1 h at 1000 rpm, at a 100:4:4:4 ratio and a solid content of 50.5% *w*/*w*. The preparation of the electrodes was performed on aluminum foil (20 μm, Schlenk SE, Roth, Germany) using a roll-to-roll coater (KTFS, Mathis AG, Oberhasli, Switzerland) with a knife coating device and two integrated convection-drying stages set to 80 and 120 °C, respectively. Drying time was less than 10 min. Compaction of the electrodes was performed in a heated calender (Saueressig GLK 200, Matthews Europe GmbH, Vreden, Germany) at 50 °C. Alternatively, a waterborne route was prepared with sodium carboxy methylcellulose, (CMC, CRT2000 PA, IFF Inc., Bomlitz, Germany) and a fluorine acrylic copolymer latex (TRD202A, JSR Micro NV., Leuven, Belgium) in a 1:1 ratio instead of the PVDF binder. The CMC solution was added at the beginning of the mixing process, whereas the latex binder was added 5 min before the end of mixing at 500 rpm. Drying temperatures for the aqueous route were 40 °C and 80 °C. 

Drying experiments of the cathodes were carried out as a discontinuous process. The aluminum foil was attached to a temperature-controlled plate. The coating was applied with a doctor blade (ZUA 2000.60, Zehntner GmbH, Sissach, Switzerland) and immediately the coating was moved under the drying nozzles of an impingement dryer. For homogeneous drying, the coating is periodically moved under the dryer until the electrode was dry.

The viscosity of the slurries was measured by a rotation viscometer (Physica MCR 101, Anton Paar GmbH, Graz, Austria) in a plate–plate configuration with a 25 mm diameter at 25 °C. The gap size was fixed at 500 µm. Before the frequency-dependent oscillation test, an amplitude sweep was performed to determine the linear viscoelastic range.

A 90° peel test, based on DIN EN 28510-1, was carried out for the measurement of the adhesion strength (10 N load cell. Zwick). Strips of the dried cathodes (17 mm in width, 60 mm in length) were attached with the coated side to an adhesive strip. The sample was pressed for 2 s at a load of 200 kg and then peeled off at a constant speed of 600 mm/min. The resulting pull-off force was measured and divided by the sample width to obtain a line adhesive force.

The electrical resistance was determined with an ohmmeter (RM3544, HIOKI E.E. Corp., Nagano, Japan). Therefore, cathode disks were placed between polished copper cylinders with a diameter of 14 mm and a load of 1 kg.

Imaging of the active material and coated electrodes was performed using a field-emission scanning electron microscope (Supra 55, Carl Zeiss GmbH, Oberkochen, Germany) with an EDS detector (Ultim Extreme, Oxford Instruments, High Wycombe, UK). Cross-sections of the electrodes were prepared by ion-beam milling (EM TIC3X, Leica Microsystems GmbH, Wetzlar, Germany) using argon ions and an accelerating voltage of 6 kV at 2.2 mA gun current. From SEM micrographs, also the primary particle size distribution of the granules was determined by image analysis (ImageJ software 1.53n) based on Feret diameters. The pore size distribution of the electrodes was determined by mercury intrusion porosimetry as described in Section 3.1.

### 3.3. Cell Preparation and Testing

Swagelok-type cells were built from the cathode layers, a Whatman GF/C separator, a lithium metal anode, and 80 µL of LP30 electrolyte (1:1 *v*/*v* EC/DMC, 1 M LiPF_6_) from Sigma-Aldrich (Taufkirchen, Germany) in a glove box (MBraun Gmbh, Garching, Germany) with O_2_ and H_2_O concentrations < 0.5 ppm. For electrochemical testing, a BT2000 battery cycler from Arbin Instruments was used. Galvanostatic cycling at discharge rates of C/20, C/10, C/5, C/2, 1C, 2C, 3C, 5C, 7C, and 10C with 1C corresponding to 183 mAh/g was performed in the voltage range between 4.3 and 3.0 V, whereas the charging rate was retained at C/2 from 1C on.

For the tortuosity measurements, a symmetrical cell was assembled with two glass fiber separators (GF/C, Whatman plc, Maidstone, UK) and 200 µL of 10 mM tetrabutylammonium perchlorate TBAClO_4_ in EC:DMC (1:1 *v*/*v*) electrolyte ( VWR International GmbH, Darmstadt, Germany). EIS measurements were performed in a temperature-controlled chamber (BTZ-175, Espec) at 25 °C using a coin cell holder (Dual CR2032 Coin Cell Holder, Gamry Instruments) with a potentiostat (VSP-300, Biologic SAS, Seyssinet-Pariset, France). The measuring parameters were 10 mV perturbation, a frequency range from 200 kHz to 100 mHz and 20 points per decade. The obtained EIS data were plotted and fitted with an EIS software (RelaxIS 3.0.21.17, rhd instruments) by using the equivalent circuit model as described in [15].

Pouch cells were assembled with a cathode size of 50 × 50 mm^2^, a ceramic-coated separator foil (Separion, Litarion GmbH, Kamenz, Germany), and graphite anodes with 54 × 54 mm^2^. Electrodes and separator were dried overnight in a vacuum furnace at 130 °C and assembled in a dry room at a dew point of below −50 °C. An amount of 450 μL of LP30 (1:1 *v*/*v* EC/DMC, 1 M LiPF6) from BASF SE was added as the electrolyte for each cell. After assembly, the cells were rested for 20 h at 40 °C to facilitate complete wetting. Cell tests were performed between 3.0 V and a cutoff voltage of 4.2 V, at a constant temperature of 23 °C. The C-rates were calculated based on 155 mAh/g as the reversible capacity for NCM111. After two initial formation cycles at 0.05C, the rate capability was tested for 0.5C, 1C, 2C, and 3C in constant current (CC) mode by symmetrical charging and discharging and 10 cycles for each C-rate. After 3C, cells were cycled again at 0.5C (10 cycles) and 1C (50 cycles) to see if irreversible capacity losses had appeared. For testing the capacity retention, cycling was continued with selected cells in 2C charge (constant current–constant voltage (CCCV) charge to a 0.05C current limit) and 3C discharge rate. Additional 10 cycles at 1C(CC) separate blocks of 100 cycles of 2C/3C to enable the calculation of incremental capacity (IC) curves regularly.

## 4. Properties of the Particles

**Figure 3:**
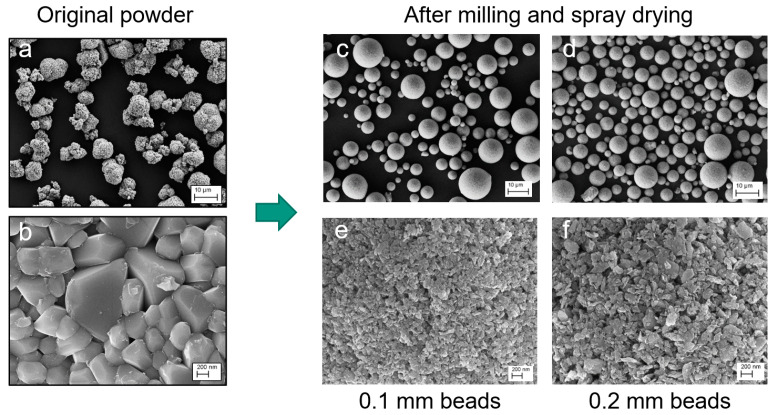
SEM micrographs of the original NCM111 particles at different magnifications (**a**,**b**), and after milling for 1 h and spray-drying (**c**,**d**). Magnifications of the spray granules show the primary particle size, which is obtained for different ZrO_2_ beads (**e**,**f**). Scale bars are 200 nm and 10 µm.

Figure 3 compares the morphology of the original NCM111 particles with the intermediate spray-dried species. The starting material has a particle size of d_50_ = 9.3 µm. The grain size is less than one micron, and the BET surface area is 0.4 m^2^/g. The milling process produces particles that are much smaller than the original material. Depending on the size of the milling beads, a particle size in the range of 100 nm (BET 49.8 m^2^/g) for 0.1 mm ZrO_2_ beads or 200 nm (BET 27.7 m^2^/g) for 0.2 mm beads is obtained after a milling time of 1 h. A particle size below 100 nm should be possible with longer milling times. The smaller particles from the 0.1 mm beads are more uniform in size, while the 0.2 mm beads also produce larger particles. Spray drying produces granules with a spherical shape, while the original particles have a more irregular shape. The size of the granules in Figure 3 is d_50_ = 7.1 µm for (c) or d_50_ = 8.6 µm for (d).

**Figure 4:**
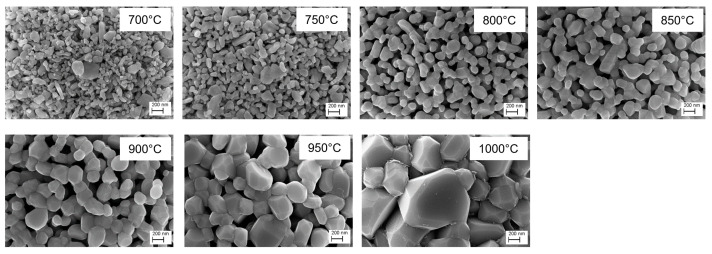
SEM micrographs of the surface of NCM111 granules after calcination at various temperatures and a dwell time of 5 h. Milling was performed with 0.2 mm ZrO_2_ beads. Scale bar is 200 nm.

The calcination process causes recrystallization and grain growth of the particles (Figure 4). Sinter necks are created, and the primary particles combine to form a secondary structure with improved mechanical stability. Residual porosity between the primary particles can be seen below a calcination temperature of 1000 °C.

**Figure 5:**
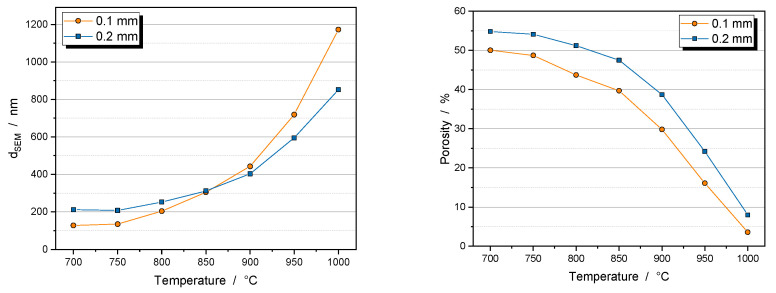
Effect of calcination temperature on primary particle size (**left**) and internal porosity of the granules (**right**). Dwell time was 5 h. Primary particles were milled with 0.1 mm beads (orange dots) or 0.2 mm beads (blue squares).

The size of the primary particles was determined from SEM images using ImageJ software. Figure 5 (left) shows that the primary particles of the 0.1 mm beads are smaller only at low calcination temperatures because small particles have a higher sintering activity than larger ones. This results in grain growth that exceeds the primary particle size of the 0.2 mm bead sample at temperatures above 850 °C. The porosity of the granules from the 0.1 mm bead sample is 5% lower in the initial state due to the better packing of the more homogeneous particle shape. During calcination, the porosity of the granules, as determined by a mercury porosimeter, decreases equally for both samples, so that the 0.1 mm bead sample has a lower porosity over the entire temperature range (Figure 5 right). Unlike the primary particles, the size of the granules changes less than 20% during the calcination step.

Despite intensive material processing, no chemical or structural changes are usually induced by the synthesis process. X-ray diffraction patterns of the original and synthesized materials show no evidence of structural changes [7]. Chemical analysis by inductively coupled plasma optical emission spectroscopy (ICP-OES) confirms that the composition of the synthesized particles is very close to the original composition [7], even after the intensive contact with water, which usually leaches measurable amounts of Li from cathode materials [16,17,18]. An explanation is that during spray drying of the milling suspension, the leached ions are retained and re-deposited on the primary particles. The original composition and phase structure are restored by solid-state diffusion processes during the subsequent heat treatment.

**Figure 6:**
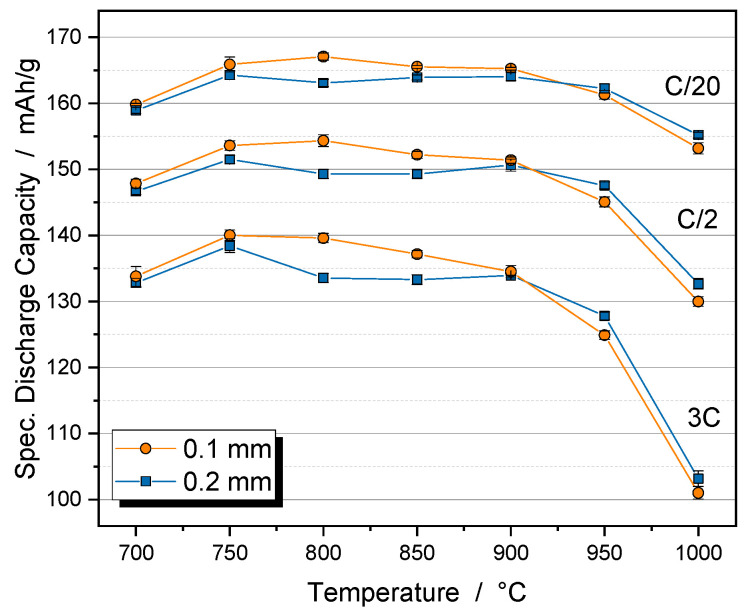
Specific discharge capacity of porous NCM111 particles depending on the calcination temperature. Primary particles were prepared with 0.1 mm beads (orange dots) or 0.2 mm beads (blue squares). Measurement in coin cells with lithium metal as the counter electrode.

The effect of the calcination temperature on the electrochemical properties of the porous NCM111 particles was investigated in coin cells against a lithium anode (Figure 6). The calcination step improves the specific discharge capacity up to a temperature of 750 °C due to the better electrical conductivity between the primary particles caused by the sinter neck formation. However, the advantages of the nanoparticles are lost as the crystallite size increases. This becomes apparent at temperatures above 900 °C, where the capacity drops significantly.

## 5. Processing of Hierarchically Structured Particles

The manufacturing processes for electrodes from hierarchically structured materials are similar to those of dense materials of the same particle size. However, there are some specific features that have a significant impact on the processing and properties of the electrodes. They occur in almost all process steps because the additional porosity within the particles affects the slurry processing, resulting in a modified additive distribution.

The hierarchically structured NMC111 particles used in the following experiments were prepared using 0.2 mm milling beads and a calcination temperature of 850 °C.

### 5.1. Slurry Mixing

**Figure 7:**
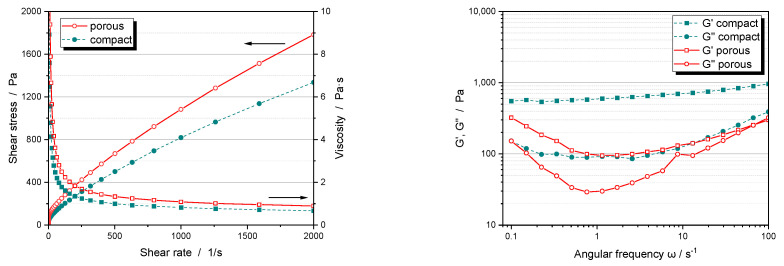
Flow and viscosity curves (**left**) and frequency dependent oscillation measurement (**right**) of NCM111 cathode slurries with dense or porous particles at a solid content 50.5 wt%.

Slurries with porous particles have a higher viscosity than those with dense particles, even though the same equipment and procedures were used for dispersing and solvent mixing (Figure 7). This is because the pores within the particles result in a higher overall porosity of 65–70 vol%. In comparison, uncalendered electrodes made from dense NCM111 particles have a porosity of only 50–55 vol% after drying. Due to the good solvent wetting and the high capillary forces created by the fine internal pores, the porous particles are completely infiltrated by the solvent. This infiltrated solvent acts as a stationary phase, reducing the available volume of the transport fluid, which in turn increases the efficient volume fraction of the particles and the internal friction in the slurry. Fluid loss remains at all shear rates, and although the viscosity curves show comparable shear thinning behavior, they therefore differ significantly up to high shear rates.

A reduction in the transport fluid between the porous particles alone should increase the binder concentration and its interactions. However, frequency-dependent oscillation measurements on the slurries show a general decrease in storage and loss modulus G’ and G”, which is particularly pronounced in the mid-frequency range (Figure 7 right). This suggests that a significant amount of the binder is also penetrating the pores, reducing the strength of the gel network. Results confirming this conjecture are presented in the following sections.

### 5.2. Drying Behavior

**Figure 8:**
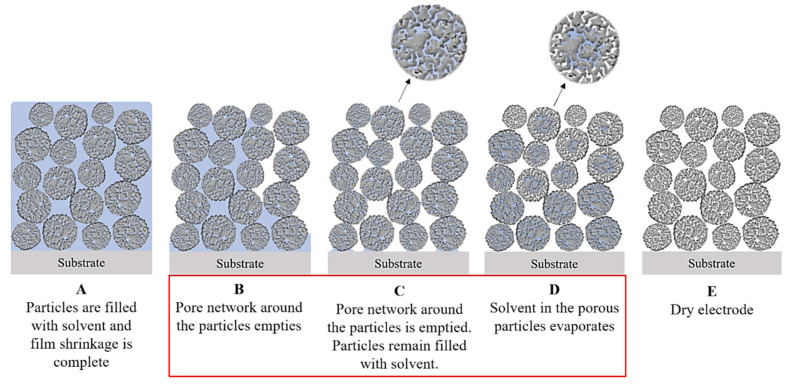
Schematic representation of the drying process of a particulate electrode with porous, nanostructured particles (Reprinted from Ref. [19] under CC BY license).

The drying behavior of the electrodes is influenced by the additional pore space of hierarchically structured materials, as described in detail by Klemens et al. [19]. At the beginning of the process, drying is similar to that of dense particles. Initially, all pores are filled with solvent (Figure 8). As solvent removal begins, the particles move closer together until they touch and film shrinkage ends. Next, large pores between the particles begin to empty. For dense particles, the drying process ends when all interparticle pores are empty; however, intragranular pores may still contain solvent because drying within the particles is slowed by the higher capillary pressure in smaller pores. An additional drying stage is required, which begins when all interparticle pores surrounding a discrete particle are empty. An evaporation front then gradually moves into the particles. It is hypothesized that this front moves homogeneously due to the narrow pore size distribution within the secondary particles, resulting in slight capillary pressure inhomogeneities. The drying process is completed when the solvent is removed from within the secondary particles.

**Figure 9:**
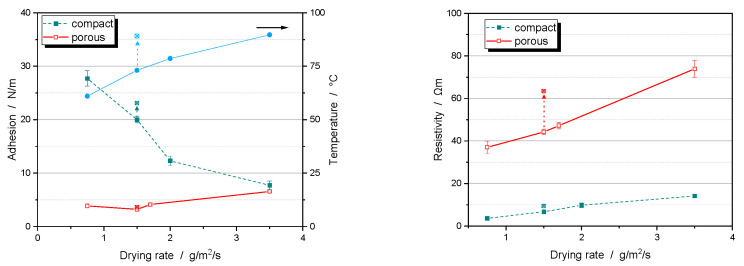
Adhesion force (**left**) and resistivity (**right**) of NCM111 cathodes with compact (green filled squares) and porous particles (red open squares) as a function of the drying rate. Also shown is the corresponding film temperature during drying (blue dots, **left**). Crossed markers indicate the results when a lower heat transfer coefficient is applied, resulting in a higher film temperature.

When electrodes are dried at higher rates, the binder may migrate to the surface of the electrode along with the solvent. This migration reduces the amount of binder at the interphase of the electrode with the current collector and decreases the adhesion [20]. This effect is particularly noticeable for compact particles (Figure 9 left). For porous particles, however, adhesion forces are generally lower and less affected by the drying rate. Interestingly, as the drying rate increases for porous particles, the adhesion forces can actually increase, bringing them closer to those of compact particles. 

As the drying rate increases, the electrical resistance of the electrodes increases. This effect occurs for both compact and porous particles (Figure 9 right). However, it is also less pronounced for porous particles. By lowering the heat transfer coefficient, the film temperature can be increased without changing the drying rate. For compact particles, this results in a significant increase in adhesion and electrical resistance. For porous particles, however, there is virtually no change in these properties. In general, porous particles appear to be more tolerant to the drying conditions than compact particles. An explanation for this behavior will be discussed in the upcoming Section 5.3 on binder distribution.

### 5.3. Additive Distribution

**Figure 10:**
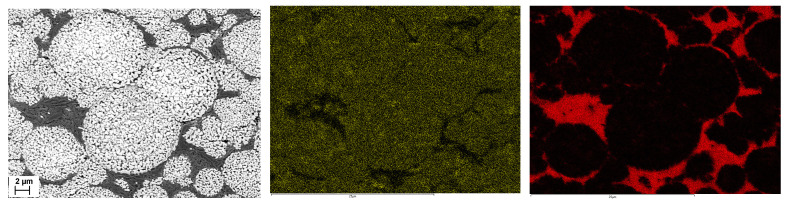
SEM micrograph of an electrode with porous NCM111 particles (**left**). Corresponding EDS mapping shows fluorine (**center**) and carbon (**right**). Images have identical magnification (Scale bar 2 µm).

If the size of the additives is smaller than the pore opening between the primary particles, they can enter the pore network of the secondary particles together with the solvent. The oscillation measurements in Section 5.1 have already indicated that this is likely to be the case for a PVDF binder. EDS mapping of the secondary particle cross-sections does indeed show the presence of fluorine, which is used as a marker for the PVDF, both around and within the secondary particles (Figure 10 center). On the other hand, no carbon can be detected inside the secondary particles (Figure 10 right). Therefore, penetration of the conductive additives used, carbon black and graphite, has not occurred.

Polymers form a compliant random coil structure whose dimensions depend on their interaction with the solvent. For example, the z-average radius of gyration of PVDF binder molecules in NMP, is in the range of 100 nm and less [17]. This allows them to enter the pores of the hierarchically structured NMC111 particles studied, which have a mean pore entry size of about 150 nm (see Section 5.4). Carbon black particles typically consist of aggregates of chemically bound primary particles, here with an average diameter of 150 nm for the Super C65 additive used, expressed as the Stokes diameter [8]. This should theoretically allow a minor fraction to penetrate the pores of the secondary particles too. However, carbon black aggregates are always associated with the PVDF to form so-called carbon black-binder domains (CBDs) [18]. The size of these domains depends on the length of the polymer chains and on the efficiency of the dispersion process. In the slurry used, the CBDs are so large that they are prevented from entering the pores of the hierarchically structured particles. As a result, the carbon black and graphite particles, which are also too large, remain in the interparticular pores around the secondary particles and do not penetrate them.

In a typical PVDF slurry formulation, only a fraction of the binder is bound to the surface of the active material or in a CBD. There is also dissolved binder that is not fixed to the surface of a particle. It is this free binder that enters the intragranular pores of the secondary particles, where it remains after drying. In this position it does not contribute to the cohesion between the particles or to the adhesion to the current collector, which explains the reduced adhesion strength of these electrodes (Figure 9 left). Without an additional binder to compensate [21], low adhesion can cause delamination between the additives, the CAM, and the aluminum foil increasing the contact resistance within the electrode [15,22]. The unbound portion of the binder is also responsible for the binder migration as described in Section 5.2, whereas a fixed binder has a limited mobility and is therefore little affected by high drying rates. In fact, binder migration also seems to occur when the porous particles empty in the second phase of the drying process (Figure 8D). With more intense drying, it appears that some of the incorporated binder migrates out, increasing the adhesion strength again. Therefore, at a sufficiently high drying rate, the loss of binder should no longer have a negative effect compared to dense particles, where a larger fraction of the binder is prone to binder migration. In this case, an additional benefit could be derived from pore infiltration as the migrated binder accumulates on the electrode surface, where it blocks ion transport. By fixing the binder in and around the particles, this negative effect can be eliminated and an improved rate capability is observed for porous particles [19].

In waterborne formulations, the situation is more complex because a two-component binder system is typically used. A dissolved polymeric binder such as carboxymethylcellulose (CMC) is augmented by a latex binder dispersion. The size of the latex particles is typically 100–200 nm. This may be small enough to allow pore penetration. However, penetration is hindered because the latex particles easily tend to clog the pore entrances. Since the CMC has the higher affinity for carbon black, virtually all of it is bound in the CBD. Therefore, in aqueous processing, the binder is located in the interparticular pores and hardly any binder is found in the intragranular pores.

### 5.4. Compaction Behavior

**Figure 11:**
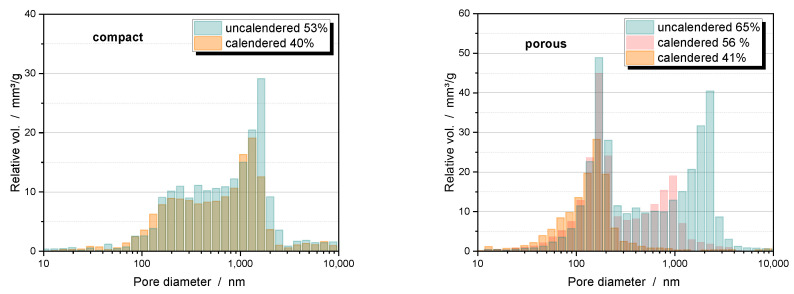
Pore size distribution of uncalendered and calendered electrodes of compact particles (**left**) and hierarchically structured NCM111 particles (**right**) at different porosities. The binder used was PVDF.

Mercury intrusion studies of uncalendered electrodes with hierarchically structured NMC111 particles reveal the existence of two distinct pore fractions (Figure 11 right). Micron-sized pores belong to the interparticular voids that are also present in electrodes made of dense particles of the same size (Figure 11 left). Calendering reduces the size of these pores only moderately for compact particles, but very effectively for porous particles, and can eliminate them completely at high densification. The pronounced pore fraction of porous particles at about 100 nm is due to the intragranular porosity. Unlike interparticular pores, intragranular pore diameters are not reduced by intensive calendering. A slight decrease in volume indicates that the pore entrance of some of the particles seems to be blocked. The broad pore fraction below 1000 nm belongs to the pores in the CBD. Moderate compression to 40% changes the porosity of the CBD only slightly for compact particles (Figure 11 left). In the case of porous particles, more densification is required to achieve a comparable overall porosity. The CBD are also densified, so that the large pores within the CBD disappear leaving mainly small pores with pore size smaller than the intragranular pores.

**Figure 12:**
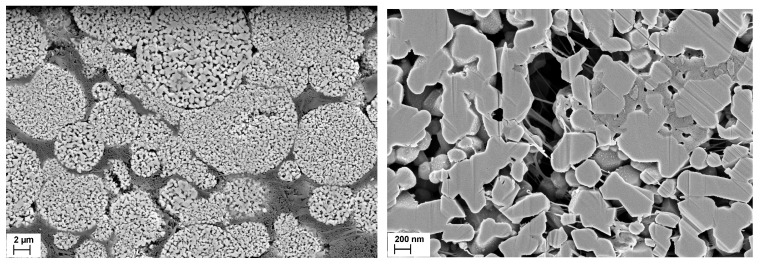
Cross-section of an electrode with deformed, hierarchically structured NMC111 particles after calendering to ~40% porosity (**left**, scale bar 2 µm). Magnification with PVDF fiber bridges within the secondary particles proofing binder penetration (**right**, scale bar 200 nm).

The development of pores during calendering is the result of a unique consolidation behavior of hierarchically structured particles. While compact particles are destroyed by high compaction forces [23], the porous particles can sometimes be deformed without defragmentation (Figure 12, left) [21]. As a result, they can be plastically compacted until the interparticular pores are eliminated and only the plastically deformable CBD remain in the voids between the secondary particles [15]. This behavior is due to PVDF bridges formed by the binder phase within these particles (Figure 12, right). They keep the particles together after the sinter bridges between the primary particles are broken and provide structural integrity. In waterborne formulations, this effect is not observed because the binders have less tendency to penetrate the secondary particles.

The tendency for plastic deformation appears to correlate with the primary particle size. Granules that deform more than their neighbors consist of finer primary particles and have weaker sinter bridges. The most likely cause of different primary particle sizes is an inhomogeneous temperature profile within the calcination furnace. As a result, grain growth and sinter neck formation are less pronounced in colder regions, resulting in granules that are less rigid.

**Figure 13:**
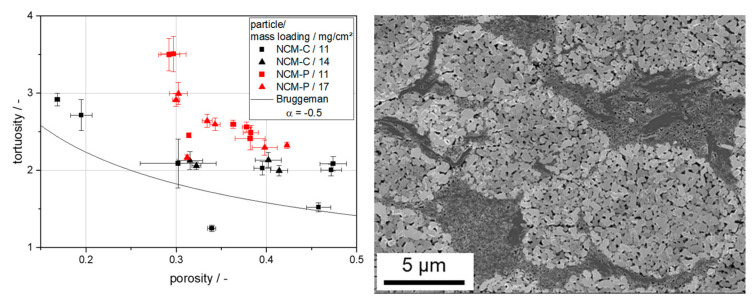
Tortuosity trends during calendering of electrodes with compact or porous NCM111 (**left**). Electrode cross-section with porous particles and compacted CBD at a porosity of 30%. The CBD contains carbon black and graphite particles (**right**) (Reprinted from Ref. [15] under CC-BY-4.0 license).

The compaction of the electrode reduces the electrical resistance and increases the ionic resistance within the pore structure [15]. While the electrical resistance of compact and porous particles exhibits similar behavior, there are clear differences in ionic resistance. Investigations using electrochemical impedance spectroscopy (EIS) show that the ionic resistance, and from this the tortuosity of the electrode, only increases moderately for compact particles (Figure 13 left), because the continuous system of interparticular pores used for ion transport remains nearly intact even at high densification. With porous particles, these pores can be eliminated due to the deformability of the particles until only the intragranular porosity and the pores within the CBD remain (Figure 13 right). The intragranular porosity is quite resistant to further densification. The pores in the CBD can be further compacted until they are also closed. However, this prevents the transport of ions through the CBD and the transport paths for the ions are considerably extended. The effect is further enhanced by graphite platelets, which are often added as an additive to improve the electrical conductivity and the compaction behavior of the electrodes. (Figure 13 right). The critical porosity at which this occurs, and at which the tortuosity increases significantly, is well above the minimum porosity that can be achieved with compact particles, although a much higher degree of compaction is required to get the porous particles to this point. Further densification of the pore structure leads to a large increase in tortuosity, and thus to higher ionic resistance, which affects the electrochemical properties of the cell. Therefore, a higher overall electrode porosity must be accepted when using porous particles. Although the intragranular porosity can still be used as a network for lithium-ion transport, the clogging of the CBD prevents an efficient contribution of these pores to the ion transport conditions in the electrode.

## 6. Electrochemical Properties

**Figure 14:**
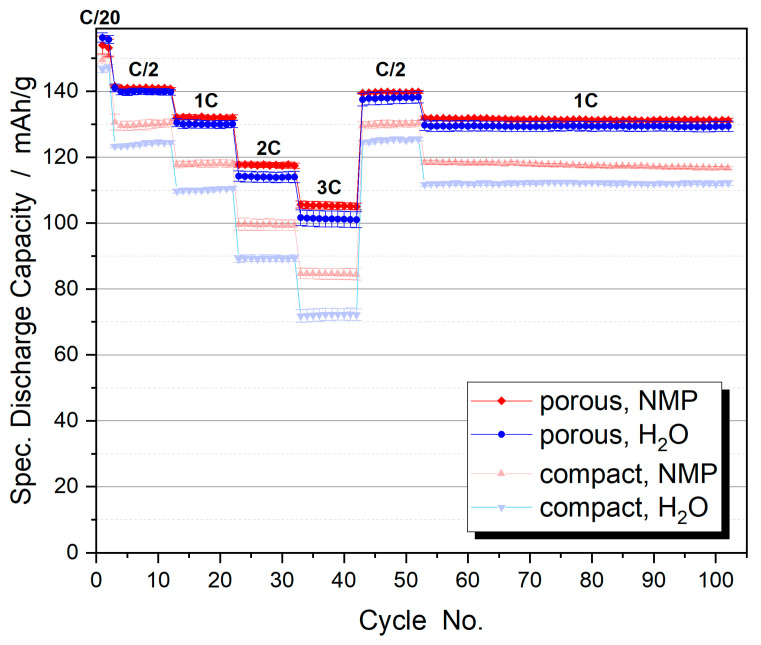
Specific discharge capacities of full cells with varying C-rates for compact NCM111 and hierarchically structured porous NCM111 particles (symmetrical CC charging). Slurry processing was carried out with NMP (red) or H_2_O (blue). Electrode porosity is 45%.

Hierarchically structured cathodes with porous NCM111 particles show significantly improved rate capability compared to the pristine dense material, when cycled against graphite anodes in pouch cell configuration (Figure 14). This is particularly evident at higher C-rates where the cells can benefit from the shortened solid-state diffusion length. A hierarchically structured half-cell model, which allows the discussion of the local lithium concentration distribution in the solid phase, shows that nanostructured secondary particles benefit not only from the reduced diffusion length but also from a more homogeneous lithium concentration distribution at higher C-rates. In this way, the available active material capacity can be better utilized, which also leads to improved performance [24]. The model also states that the rate-limiting factor for cathodes with dense particles is the diffusion coefficient of the active material, whereas it plays a minor role for hierarchically structured cathodes. Here, the combination of electronic conductivity and connectivity between the primary particles is rate-limiting. Interestingly, an improved capacity can be observed even in the slow formation cycles at C/20. Under these conditions, the electrodes do not benefit from the kinetic advantages of the small primary particles. However, the significantly higher electrochemically active area of the porous material also reduces the charge transfer resistance [5] and decreases the activation overpotential at the particle–electrolyte interphase, leading to a reduced potential rise during charging [25]. In summary, at low C-rates a higher electrochemically active area with reduced phase transfer resistance contributes to the superior capacity, while at high C-rates the shorter diffusion pathways are the main advantage of hierarchically structured electrodes.

**Figure 15:**
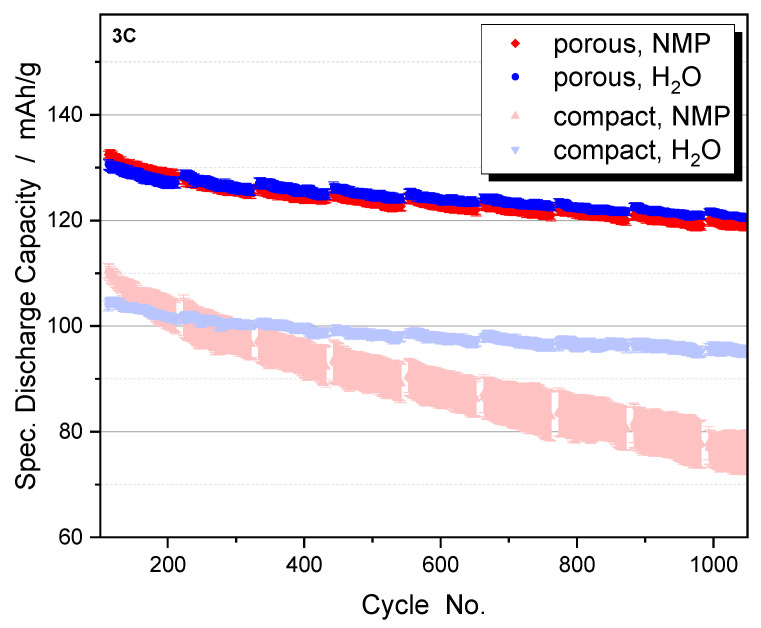
Aging behavior of compact NCM111 and hierarchically structured porous NCM111 particles (CCCV charging at 2C, 3C discharging). Slurry processing was carried out with NMP (red) or H_2_O (blue). Electrode porosity is 45%.

**Figure 16:**
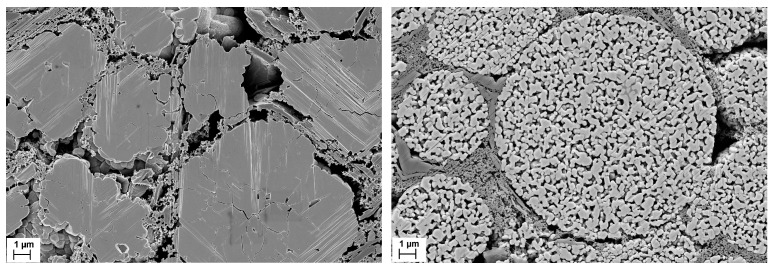
SEM micrographs of electrode cross-sections after 1100 cycles of compact (**left**) and porous (**right**) NCM111 particles. Processing was carried out with NMP/PVDF slurry (scale bars are 1 µm).

The advantages of nanoscale particles are also evident in the aging behavior of the cycled electrodes (Figure 15). The hierarchically structured electrodes show a significantly slower capacity decrease than the electrodes with compact particles. This is probably due to the state of the aged active material particles [21,22]. While the micro-sized compact particles show numerous cracks due to the mechanical stresses generated during cycling, the hierarchically structured particles do not show any large cracks (Figure 16). As expected, the small size of the primary particles leads to lower stress numbers and thus reduces the formation of cracks. The secondary particle structures are not destroyed after more than 1000 cycles. Where cracks occur between the primary particles, the contact is not broken because the two particles can still be held together by binder bridges. In large compact particles, crack formation causes a portion of the particles to lose electrical connectivity resulting in a significant loss of active material over the course of cycling [26].

An interesting result is found for the aqueous processing of the electrodes. One would expect that the increased surface area of the porous particles leads to more interaction with water, resulting in a decrease in capacity and increased degradation. In fact, aqueous processing of hierarchically structured electrodes with porous NCM111 particles results in a rate capability (Figure 14, blue dots) and aging behavior (Figure 15, blue dots) similar to that obtained when NMP is used as the solvent. In contrast, dense NCM111 particles show a significant capacity loss in water, although they do not show extensive aging. Specific advantages of the hierarchically structured electrodes seem to compensate for a stronger interaction with water. For example, a cleaning process could take place during the synthesis of the porous particles or structural defects could heal during calcination. These effects should be the subject of future investigations.

## 7. Outlook: Post-Lithium Batteries

Post-lithium batteries using sodium, potassium, magnesium, aluminum, etc., as active elements are of particular interest as low-cost and sustainable alternatives to lithium-ion batteries [27,28,29]. These systems offer interesting applications for hierarchically structured materials. Due to the larger ions, intercalation/deintercalation leads to larger stresses in the material. Multivalent ions have extremely low diffusion coefficients allowing only a small penetration depth. Therefore, the use of a nanoscale material is necessary to ensure high energy densities and sufficient lifetime, and hierarchically structured particles may be the only chance to establish these materials in commercial applications.

**Figure 17:**
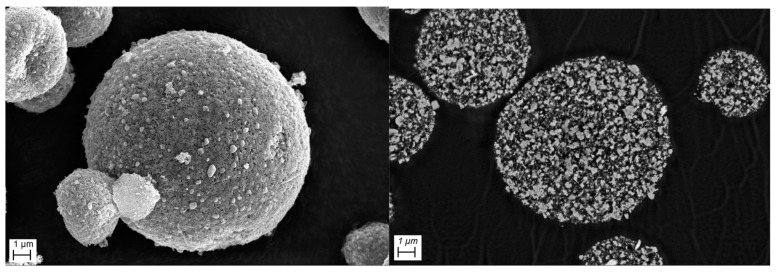
SEM micrograph of NVP/C composite particles (**left**), corresponding cross-sections (**right**). Scale bars are 1 µm.

An example of a sodium-ion battery material is Na_3_V_2_(PO_4_)_3_ (NVP). It features a highly stable three-dimensional NASICON structure and offers large Na diffusion pathways [30]. However, like all sodium super-ionic conductors it has remarkably low electronic conductivity [31]. To make this material suitable for batteries, it is either necessary to reduce the particle size [32,33] or to combine it with a conductive carbon matrix [34]. The preparation of a hierarchically structured material makes it possible to combine both approaches in a single particle system. SEM micrographs of nanoporous composite particles consisting of an NVP matrix with an embedded carbon phase and an intrinsic porosity of up to 54% are shown in Figure 17. They were synthesized via a bottom-up approach and show excellent cycling stability when used as either a cathode or anode material [35] or within full cells [36].

## 8. Summary

Hierarchically structured electrode materials combine the advantages of nanomaterials in terms of ion transport and lower susceptibility to mechanically induced defects with the better packing density of micro-sized particles. For this purpose, nanoscale primary particles are granulated to form secondary structures at the microscale. A calcination step is necessary to produce mechanically stable particles with an open pore phase that can be processed similarly to established electrode materials. Electrodes made from this material have a hierarchically structured porosity, i.e., in addition to the usual microscale interparticular pores, there are nanoscale intragranular pores that can be penetrated by the electrolyte. The expected advantages of this setup are confirmed by the improved rate capability and lifetime of the electrodes. Since only short distances need to be bridged by means of solid-state diffusion, high capacities can be achieved even at high C-rates. The increased specific surface area also reduces the phase transfer resistance, which increases the charge capacity even at low C-rates. The small size of the primary particles results in less mechanical stress due to the volume changes during cycling, which increases cell life by reducing particle cracking. Benefits have also been demonstrated for the aqueous processing of cathode materials, as no additional material degeneration is observed despite the increased reaction area with water, and cycling performance is comparable to processing with NMP.

The advantages of nanostructured particles and electrodes are partially offset by the disadvantages of increased additive requirements and reduced energy density. The open pore phase can absorb some of the additives, which then no longer provide the intended effects and must be replaced, for example by additional binder to ensure equivalent adhesion. However, the infiltrated binder has the advantage of holding the primary particles together, even if cracks form in the porous secondary particles during calendering. As a result, the particles have a quasi-plastic deformation behavior that enables complete elimination of interparticle porosity until only intragranular porosity and the pores in the CBD remain in the electrode. Further densification also occludes the CBD and blocks them to the ions. Although the residual porosity within the secondary particles is still higher than the porosity level achievable by calendering compact particles, the transport paths lengthen and the ion resistance in the electrode increases significantly so that the advantage of the open particle porosity for ion transport is lost. 

The hierarchically structured materials approach is therefore of particular interest for active materials with low electrical or ionic conductivity. Commercial lithium iron phosphate (LFP) materials often have particles with a composite structure and internal porosity. Other examples are fluoride-based cathode materials or many active materials for post-lithium systems, which cannot achieve near-application energy densities without a massively reduced particle size. While improved rate capability and reduced aging are offset by lower energy density, it is important to consider whether the additional effort in particle synthesis is worthwhile. Nevertheless, it is often only hierarchically structured electrodes that enable the use of such materials in competitive cells.

## Data Availability

Data that support the findings are available from the corresponding author upon reasonable request.

## References

[1] Meng Z., Ma X., Azhari L., Hou J., Wang Y. (2023). Morphology controlled performance of ternary layered oxide cathodes. Commun. Mater..

[2] Ren D., Padgett E., Yang Y., Shen L., Shen Y., Levin B.D.A., Yu Y., Disalvo F.J., Muller D.A., Abruña H.D. (2019). Ultrahigh Rate Performance of a Robust Lithium Nickel Manganese Cobalt Oxide Cathode with Preferentially Orientated Li-Diffusing Channels. ACS Appl. Mater. Interfaces.

[3] Jiang M., Zhang Q., Wu X., Chen Z., Danilov D.L., Eichel R.A., Notten P.H.L. (2020). Synthesis of Ni-Rich Layered-Oxide Nanomaterials with Enhanced Li-Ion Diffusion Pathways as High-Rate Cathodes for Li-Ion Batteries. ACS Appl. Energy Mater..

[4] Yang Z., Lu J., Bian D., Zhang W., Yang X., Xia J., Chen G., Gu H., Ma G. (2014). Stepwise co-precipitation to synthesize LiNi_1/3_Co_1/3_Mn_1/3_O_2_ one-dimensional hierarchical structure for lithium ion batteries. J. Power Sources.

[5] Lin B., Wen Z., Gu Z., Huang S. (2008). Morphology and electrochemical performance of Li[Ni_1/3_Co_1/3_Mn_1/3_]O_2_ cathode material by a slurry spray drying method. J. Power Sources.

[6] Oljaca M., Blizanac B., Du Pasquier A., Sun Y., Bontchev R., Suszko A., Wall R., Koehlert K. (2014). Novel Li(Ni_1/3_Co_1/3_Mn_1/3_)O_2_ cathode morphologies for high power Li-ion batteries. J. Power Sources.

[7] Wagner A.C., Bohn N., Geßwein H., Neumann M., Osenberg M., Hilger A., Manke I., Schmidt V., Binder J.R. (2020). Hierarchical Structuring of NMC111-Cathode Materials in Lithium-Ion Batteries: An In-Depth Study on the Influence of Primary and Secondary Particle Sizes on Electrochemical Performance. ACS Appl. Energy Mater..

[8] Deng W.N., Li Y.H., Xu D.F., Zhou W., Xiang K.X., Chen H. (2022). Three-dimensional hierarchically porous nitrogen-doped carbon from water hyacinth as selenium host for high-performance lithium–selenium batteries. Rare Met..

[9] Naumann J., Bohn N., Birkholz O., Neumann M., Müller M., Binder J.R., Kamlah M. (2023). Morphology-Dependent Influences on the Performance of Battery Cells with a Hierarchically Structured Positive Electrode. Batter. Supercaps.

[10] Chen D., Kramer D., Mönig R. (2018). Chemomechanical fatigue of LiMn_1.95_Al_0.05_O_4_ electrodes for lithium-ion batteries. Electrochim. Acta.

[11] Spahr M.E., Goers D., Leone A., Stallone S., Grivei E. (2011). Development of carbon conductive additives for advanced lithium ion batteries. J. Power Sources.

[12] Bockholt H., Haselrieder W., Kwade A. (2016). Intensive powder mixing for dry dispersing of carbon black and its relevance for lithium-ion battery cathodes. Powder Technol..

[13] McLachlan D.S., Blaszkiewicz M., Newnham R.E. (1990). Electrical Resistivity of Composites. J. Am. Ceram. Soc..

[14] Radin M.D., Hy S., Sina M., Fang C., Liu H., Vinckeviciute J., Zhang M., Whittingham M.S., Meng Y.S., Van der Ven A. (2017). Narrowing the Gap between Theoretical and Practical Capacities in Li-Ion Layered Oxide Cathode Materials. Adv. Energy Mater..

[15] Schneider L., Klemens J., Herbst E.C., Müller M., Scharfer P., Schabel W., Bauer W., Ehrenberg H. (2022). Transport Properties in Electrodes for Lithium-Ion Batteries: Comparison of Compact versus Porous NCM Particles. J. Electrochem. Soc..

[16] Loeffler N., Kim G.T., Mueller F., Diemant T., Kim J.K., Behm R.J., Passerini S. (2016). In Situ Coating of Li[Ni_0.33_Mn_0.33_Co_0.33_]O_2_ Particles to Enable Aqueous Electrode Processing. ChemSusChem.

[17] Lutringer G., Weill G. (1991). Solution properties of poly(vinylidene fluoride): 1. Macromolecular characterization of soluble samples. Polymer.

[18] Wood M., Li J., Ruther R.E., Du Z., Self E.C., Meyer H.M., Daniel C., Belharouak I., Wood D.L. (2020). Chemical stability and long-term cell performance of low-cobalt, Ni-Rich cathodes prepared by aqueous processing for high-energy Li-Ion batteries. Energy Storage Mater..

[19] Klemens J., Schneider L., Herbst E.C., Bohn N., Müller M., Bauer W., Scharfer P., Schabel W. (2022). Drying of NCM Cathode Electrodes with Porous, Nanostructured Particles Versus Compact Solid Particles: Comparative Study of Binder Migration as a Function of Drying Conditions. Energy Technol..

[20] Zhang Y.S., Courtier N.E., Zhang Z., Liu K., Bailey J.J., Boyce A.M., Richardson G., Shearing P.R., Kendrick E., Brett D.J.L. (2022). A review of lithium-ion battery electrode drying: Mechanisms and metrology. Advanced Energy Materials.

[21] Muller M., Schneider L., Bohn N., Binder J.R., Bauer W. (2021). Effect of Nanostructured and Open-Porous Particle Morphology on Electrode Processing and Electrochemical Performance of Li-Ion Batteries. ACS Appl. Energy Mater..

[22] Dreizler A.M., Bohn N., Geßwein H., Müller M., Binder J.R., Wagner N., Friedrich K.A. (2018). Investigation of the Influence of Nanostructured LiNi_0.33_Co_0.33_Mn_0.33_O_2_ Lithium-Ion Battery Electrodes on Performance and Aging. J. Electrochem. Soc..

[23] Oswald S., Pritzl D., Wetjen M., Gasteiger H.A. (2020). Novel Method for Monitoring the Electrochemical Capacitance by In Situ Impedance Spectroscopy as Indicator for Particle Cracking of Nickel-Rich NCMs: Part I. Theory and Validation. J. Electrochem. Soc..

[24] Birkholz O., Kamlah M. (2021). Electrochemical Modeling of Hierarchically Structured Lithium-Ion Battery Electrodes. Energy Technol..

[25] Lu X., Daemi S.R., Bertei A., Kok M.D.R., O’Regan K.B., Rasha L., Park J., Hinds G., Kendrick E., Brett D.J.L. (2020). Microstructural Evolution of Battery Electrodes During Calendering. Joule.

[26] Ryu H.H., Park N.Y., Noh T.C., Kang G.C., Maglia F., Kim S.J., Yoon C.S., Sun Y.K. (2021). Microstrain Alleviation in High-Energy Ni-Rich NCMA Cathode for Long Battery Life. ACS Energy Lett..

[27] Kundu D., Talaie E., Duffort V., Nazar L.F. (2015). The emerging chemistry of sodium ion batteries for electrochemical energy storage. Angew. Chem. Int. Ed..

[28] Wang Y., Chen R., Chen T., Lv H., Zhu G., Ma L., Wang C., Jin Z., Liu J. (2016). Emerging non-lithium ion batteries. Energy Storage Mater..

[29] Ponrouch A., Bitenc J., Dominko R., Lindahl N., Johansson P., Palacin M.R. (2019). Multivalent rechargeable batteries. Energy Storage Mater..

[30] Song W., Ji X., Wu Z., Zhu Y., Yang Y., Chen J., Jing M., Li F., Banks C.E. (2014). First exploration of Na-ion migration pathways in the NASICON structure Na_3_V_2_(PO_4_). J. Mater. Chem. A.

[31] Lan T., Ma Q., Tsai C.L., Tietz F., Guillon O. (2021). Ionic Conductivity of Na_3_V_2_P_3_O_12_ as a Function of Electrochemical Potential and its Impact on Battery Performance. Batter. Supercaps.

[32] Jiang Y., Yang Z., Li W., Zeng L., Pan F., Wang M., Wei X., Hu G., Gu L., Yu Y. (2015). Nanoconfined carbon-coated Na_3_V_2_(PO_4_)_3_ particles in mesoporous carbon enabling ultralong cycle life for sodium-ion batteries. Adv. Energy Mater..

[33] Zheng W., Huang X., Ren Y., Wang H., Zhou S., Chen Y., Ding X., Zhou T. (2017). Porous spherical Na_3_V_2_(PO_4_)_3_/C composites synthesized via a spray drying -assisted process with high-rate performance as cathode materials for sodium-ion batteries. Solid State Ion..

[34] Jian Z., Zhao L., Pan H., Hu Y.S., Li H., Chen W., Chen L. (2012). Carbon coated Na_3_V_2_(PO_4_)_3_ as novel electrode material for sodium ion batteries. Electrochem. Commun..

[35] Akçay T., Häringer M., Pfeifer K., Anhalt J., Binder J.R., Dsoke S., Kramer D., Mönig R. (2021). Na_3_V_2_(PO_4_)_3_-A Highly Promising Anode and Cathode Material for Sodium-Ion Batteries. ACS Appl. Energy Mater..

[36] Stüble P., Müller C., Klemens J., Scharfer P., Schabel W., Häringer M., Binder J.R., Hofmann A., Smith A. (2023). Enabling Long-term Cycling Stability of Na_3_V_2_(PO_4_)_3_/C vs. Hard Carbon Full-cells. Batter. Supercaps.

